# Coexistence of two novel resistance plasmids, *bla*_KPC-2_-carrying p14057A and *tetA*(A) -carrying p14057B, in *Pseudomonas aeruginosa*

**DOI:** 10.1080/21505594.2017.1372082

**Published:** 2017-10-04

**Authors:** Lining Shi, Quanhui Liang, Jiao Feng, Zhe Zhan, Yachao Zhao, Wenhui Yang, Huiying Yang, Yong Chen, Mei Huang, Yigang Tong, Xiaojun Li, Zhe Yin, Jinglin Wang, Dongsheng Zhou

**Affiliations:** a Institute of Medical Laboratory Sciences, Jinling Hospital, School of Medicine, Nanjing University, Nanjing, China; b State Key Laboratory of Pathogen and Biosecurity, Beijing Institute of Microbiology and Epidemiology, Beijing, China; c Department of Clinical Laboratory, the First People's Hospital of Foshan, Foshan, China

**Keywords:** KPC-2, multidrug resistance, plasmid, *Pseudomonas aeruginosa*, tetA

*Klebsiella pneumoniae* carbapenamases (KPCs) are class A β-lactamases that efficiently hydrolyze almost all β-lactams including carbapenems, with exception of cephamycins. The *bla*_KPC_ genes were initially discovered in *K. pneumoniae* in 1996 and have been frequently detected all over the world in particular among the species belonging to the family *Enterobacteriaceae*.^1^ KPC-producing *Pseudomonas aeruginosa* isolates were initially found in 2006 from Colombia and increasingly being reported in South America, North America, the Caribbean islands, and China,^2,3^ indicating a potential worldwide spread of KPC-producing *P. aeruginosa*. The *bla*_KPC_ genes from *P. aeruginosa* are located on chromosome or plasmids.^4^ Up to now, five fully sequenced *bla*_KPC_-carrying plasmids from *P. aeruginosa* are available in public databases: two IncP-6 plasmids pCOL-1 (accession number KC609323) and p10265-KPC (accession number KU578314) from Colombia^5^ and China,^6^ respectively, two IncU plasmids pPA-2 (accession number KC609322) and pD5170990 (accession number KX169264) from Colombia^5^ and Brazil, respectively, and pBH6 (accession number CM003767; it could not be assigned into any of known incompatibility groups) from Brazil.^7^ Each of pCOL-1, p10265-KPC, pPA-2, and pBH6 contains the sole resistance gene *bla*_KPC-2_, while pD5170990 carries *bla*_KPC-2_, *strAB* (aminoglycoside resistance), *cmx* (chloramphicol resistance), *qacED1* (quaternary ammonium compound resistance), and *sul1* (sulphonamide resistance).

The *tet* loci responsible for inducible tetracycline resistance commonly contain a *tetA* gene encoding the transmembrane tetracycline efflux protein, and a *tetR* gene encoding the tetracycline repressor.^8^ At least 38 different classes (A, B, etc) of *tet* efflux have been identified, giving the designations *tetA*(A)-*tetR*(A), *tetA*(B)-*tetR*(B), etc.^8^ In *P. aeruginosa*, the class A tetracycline resistance module *tetA(A)-tetR(A)* has been detected in only IncP-1α plasmids, including RP1/RP4/R18/R68/RK2 (identical to each other; accession number L27758) from United Kingdom,^9^ pBS228 (accession number AM261760) from Russia,^10^ and R1033 (accession number HM804085) from Spain;^11^ among them RP1/RP4/R18/R68/RK2 and pBS228 were fully sequenced. This study dealt with detailed genomic characterization of two novel plasmids p14057A and p14057B found in a single clinical *P. aeruginosa* isolate.

On January 1st 2016, a middle-aged male with acute epigastric pain, which occurred upon nocturnal excessive drinking, was hospitalized in a local hospital in Nanjing City, China, and diagnosed to have acute pancreatitis. The symptoms of severe respiratory/renal failure and obnubilation progressed although the patient received the rehydration, acid suppression, and anti-inflammatory therapies. The patient was transferred to Jinling Hospital on January 3rd and received a series of symptomatic treatments, especially including continuous (until January 27th) and subsequent intermittent (until February 7th) mechanical ventilation, and repeated abdominal cavity drainage (until February 18th), and his symptoms associated with pancreatitis gradually improved. The patient started to suffer again from fever and respiratory difficulty since January 13th and was diagnosed to have ventilator-associated pneumonia. *P. aeruginosa* 14057 was isolated from the sputum specimens after repeated sampling and cultivation from January 13th to 15th. Bacterial species was confirmed by 16S rRNA gene sequencing^12^ and PCR detection of *P. aeruginosa*-specific *oafA* gene.^13^ According to antimicrobial susceptibility test results (see below), the patient received intravenous administration with amikacin plus ceftriaxone, and his symptoms associated with pneumonia progressively disappeared.

The major plasmid-borne carbapenemase genes were screened for by PCR,^14^ followed by amplicon sequencing on ABI 3730 Sequencer (LifeTechnologies, CA, USA). Out of all the carbapenemase genes tested, only *bla*_KPC-2_ was detected in the 14057 isolate.

Plasmid DNA was isolated from the 14057 isolate using a Qiagen large construct kit, followed by sequencing from a mate-pair library with average insert size of 5,000 bp, using a MiSeq sequencer (Illumina, CA, USA). Sequence assembly and annotation were performed as described previously.^15^ The contigs were assembled using Newbler 2.6.^16^ Open reading frames and pseudogenes were predicted using RAST 2.0^17^ combined with BLASTP/BLASTN^18^ searches against the UniProtKB/Swiss-Prot database^19^ and the RefSeq database.^20^ Annotation of resistance genes, mobile elements, and other features was carried out using online databases including CARD,^21^ ResFinder,^22^ BacMet,^23^ ISfinder,^24^ INTEGRALL,^25^ and the Tn Number Registry.^26^ Multiple and pairwise sequence comparisons were performed using MUSCLE 3.8.31^27^ and BLASTN, respectively. Gene organization diagrams were drawn in Inkscape 0.48.1.

**Figure 1.**
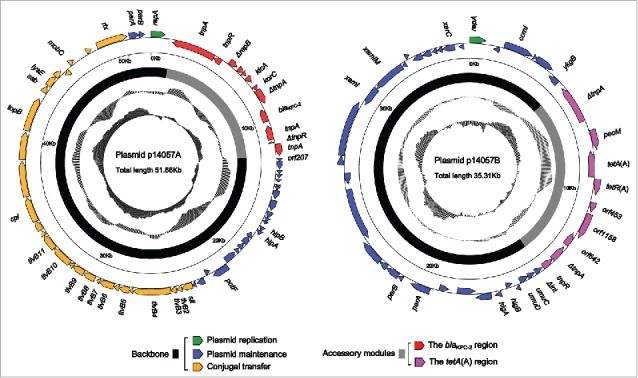
Schematic map of p14057A and p14057B. Genes are denoted by arrows, and the backbone and accessory module regions are highlighted in black and color, respectively. The innermost circle presents GC-skew [(G-C)/(G+C)], with a window size of 500 bp and a step size of 20 bp. The next-to-innermost circle presents GC content. Shown also are backbone and accessory modules.

It was revealed that the 14057 isolate harbored two circularly closed DNA sequences, designated p14057A and p14057B, which were 51,663 bp and 35,306 bp in length with mean G+C contents of 59.2% and 56.8% and contained 65 and 43 predicted ORFs, respectively (Fig. 1). The modular structure of each of p14057A and p14057B was discriminated as the backbone regions with insertion of a single accessory module, namely the *bla*_KPC-2_ region and the *tetA*(A) region, respectively (Fig. 1). The accessory modules were defined as acquired DNA regions associated with and bordered by mobile elements.

The backbones of p14057A and p14057B, 40.6 kb and 29.7kb in length, respectively, showed very low levels of similarity (≤11% query coverage) to DNA sequences available in public sequence databases. Located in the p14057A backbone were *repA* and its iterons responsible for plasmid replication initiation, *parAB* for plasmid partition, *higBA* encoding the toxin-antitoxin system for post-segregational killing, and a 27-kb conjugal transfer region that harbored *tivB* genes encoding a type IV secretion system.^28^ The p14057B backbone contained genes involved in plasmid replication initiation (*repA* and its iterons) and maintenance (*parAB, higBA, xerC, xamIM*, and *umuCD*), but lacked those for conjugal transfer. *xerC* encoded a site-specific recombinase helping to resolve DNA dimers into monomers after termination of replication.^29^ *xamIM* encoded a restriction-modification system involved in post-segregational killing.^30^ *umuCD* encoded the SOS stress-inducible DNA polymerase for DNA lesion bypass.^31^ The deduced RepA proteins of p14057A and p14057B had the highest matches (>97% amino acid similarity) to two different *P. aeruginosa* RepA proteins (accession numbers ERX63685 and WP_059309776.1), respectively. All the above replication proteins could not be assigned into any of known incompatibility groups.

**Figure 2.**
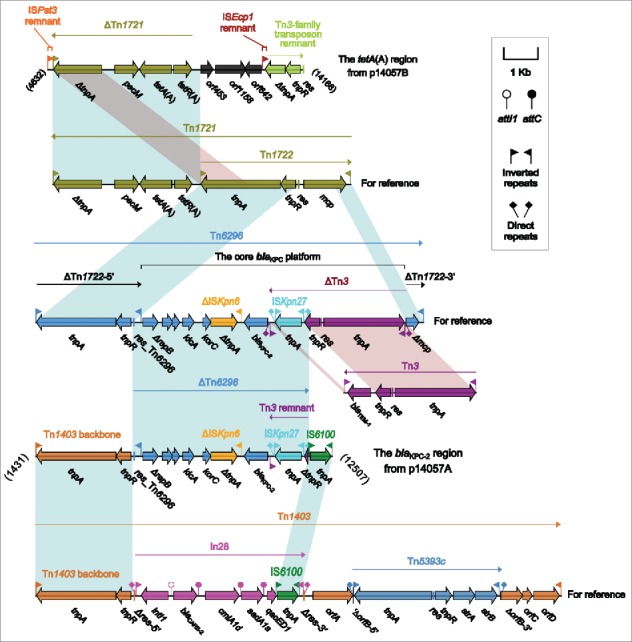
The accessory regions of p14057A and p14057B and comparison to related genetic contents. Genes are denoted by arrows. Genes, mobile elements and other features are colored based on function classification. Shading denotes regions of homology (>95% nucleotide identity). Numbers in brackets indicate the nucleotide positions within the corresponding plasmids. The accession numbers of Tn*1721*, Tn*3*, Tn*6296*, and Tn*1403* for reference are X61367, HM749966, FJ628167, and AF313472, respectively.

The *bla*_KPC-2_ region (Fig. 2) of p14057A was organized in order of a 3.5-kb partial region of the Tn*1403* backbone, a 6.5-kb ΔTn*6296* region, and an IS*6100* element. Tn*1403* was a multi-drug resistant transposon with insertion of In28 within the resolution (*res*) site and that of Tn*5393c* within *orfB*.^32^ Insertion of foreign MDR regions at the sites within *res* and other regions generated Tn*1403* derivatives such as Tn*6060*, Tn*6061*, Tn*6162* and Tn*6249* in *P. aeruginosa*.^33^ The ΔTn*3*:IS*Kpn27* to *ΔrepB* region represented a core *bla*_KPC_ platform, and its insertion into the *mcp* gene of the cryptic transposon Tn*1722* carried in Tn*1721*,^34^ leaving *mcp* to be truncated, generated the prototype Tn*6296* as observed in pKP048.^35^ Various Tn*6296* derivatives with different deletions, insertions and rearrangements had been found in KPC-encoding plasmids from China.^6,36^ In p14057A, ΔTn*6296* manifested as a partial fragment of Tn*6296* and consisted of *res* and the core *bla*_KPC_ platform with further truncation of Tn*3* (likely resulted from its connection with downstream IS*6100*), while the partial Tn*1403* backbone region was composed of the 38-bp inverted repeat left (IRL) and the core transposition module *tnpA* (transposase)-*tnpR* (resolvase) lacking *res*. Both Tn*1403* and Tn*6296* belonged to the Tn*21* subgroup of the Tn*3* family, and their terminal inverted repeats and core transposition modules shared significant nucleotide sequence homology, which might facilitate homologous recombination between Tn*1403*- and Tn*6296*-like elements, generating the *bla*_KPC-2_ region of p14057A.

The *tetA*(A) region (Fig. 2) of p14057B was organized sequentially as an 150-bp remnant of IS*Pst3*, a 5.5-kb ΔTn*1721* region, *orf453, orf1158, orf642*, a 201-bp remnant of IS*Ecp1*, and an 1.4-kb remnant of a novel Tn*3*-family transposon core transposition module. Tn*1721* was also a member of the Tn*21* subgroup of Tn*3* family, and had an unusual structure that included three 38-bp IR elements and a partial duplication of the *tnpA* gene.^34^ Tn*1722* was a independently transposable transposon with an IRL-*tnpAR-res-mcp*-IRR structure and located at the 3'-region of Tn*1721*, while the other end of Tn*1721* included the *pecM-tetA(A)-tetR(A)* region, which represented a core genetic platform of class A tetracycline resistance genes.^34^ The ΔTn*1721* region was a 3'-terminal resistance fragment of Tn*1721*,^34^ and carried the class A tetracycline resistance module *tetA(A)-tetR(A)*. This is the first report of detecting *tetA(A)* and *tetR(A)* in *P. aeruginosa* from China.

Plasmids were transferred in attempt from the 14057 isolate into *Escherichia coli* TOP10 or DH10B and EC600 (highly resistant to rifampicin) through electroporation and conjugal transfer, respectively.^15^ For selection of electroporant or transconjugant containing *tetA*(A) or *bla*_KPC_, 10 μg/ml tigecycline, 2 μg/ml imipenem, and 1000 μg/ml rifampicin were used in accordance with specific circumstances. Neither p14057A nor p14057B could be transferred into EC600 through conjugal transfer. p14057A was transferred into TOP10 through electroporation, yielding the *bla*_KPC-2_-carrying electroporant TOP10(p14057A), but p14057B could not be transferred into DH10B. Activity of Ambler class A/B/D carbapenemases in bacterial cell extracts was determined by a modified CarbaNP test.^15^ Both 14057 and TOP10(p14057A) had the activity of class A carbapenemase, which was attributable to production KPC-2 enzyme (data not shown). Bacterial antimicrobial susceptibility was tested by the broth dilution method, and interpreted as per CLSI guidelines.^37^ The 14057 isolate was resistant to ampicillin, ceftazidime, imipenem, aztreonam, azithromycin, minocycline, ciprofloxacin, trimethoprim, and sulfamethoxazole, but susceptible to amikacin, tigecycline, and colistin; TOP10(p14057A) was resistant to ampicillin, ceftazidime, imipenem, and aztreonam, but susceptible to all the other drugs tested (Table 1).

**Table 1.** Antimicrobial drug susceptibility profiles.

	MIC (mg/L)/antimicrobial susceptibility		
Antibiotics	14057	TOP10 (p14057A)	TOP10
Ampicillin	>1024/R@	512/R	<4/S
Ceftazidime	512/R	16/R	<4/S
Imipenem	32/R	8/R	<1/S
Aztreonam	>512/R	32/R	<4/S
Azithromycin	32/R@	<4/S	<4/S
Minocycline	128/R@	4/S	4/S
Ciprofloxacin	8/R	<1/S	<1/S
Trimethoprim	4/R@	<0.25/S	<0.25/S
Sulfamethoxazole	76/R	4.75/S	4.75/S
Amikacin	<8/S	<8/S	<8/S
Tigecycline	<1/S	<1/S	<1/S
Colistin	<1/S	<1#	<1#

*Note.*

S = sensitive; R = resistant; R@ = intrinsically resistant; # = clinical breakpoints non-available

p14057A appears to be potentially self-transmissible due to the fact that is harbors a potentially complete set of conjugative transfer genes. p14057B seems unlikely to be self-transmissible because it contains none of putative mobilization or conjugal transfer regions. Each of these two plasmids carries a single accessory module, namely the *bla*_KPC-2_ region and the *tetA*(A) region, respectively, and these two resistance genes serve as the sole resistance determinant of the corresponding plasmid. The core genetic environments of *bla*_KPC-2_ and *tetA(A)-tetR(A)* have been well characterized previously, but they are connected with additional transposon- and insertion sequence-like elements to generate the novel *bla*_KPC-2_ and *tetA*(A) regions inserted in p14057A and p14057B, respectively, which might be resulted from complex recombination events. These two accessory resistance regions cannot be identified as native transposition units because they lack paired terminal inverted repeats at both ends. Co-existence of two different resistance plasmids has been reported in the clinical *P. aeruginosa* isolates from India,^38,39^ but none of these plasmids were sequenced. This work presented the first two fully sequenced resistance plasmids coexisting in *P. aeruginosa*, which is a significant cause of nosocomial infection. Data presented here provides a deeper insight into how *P. aeruginosa* becomes drug-resistant by diversified mechanisms, and illustrates the way that genomic sequence analysis allows assessment of the genetic basis of plasmid-mediated resistance profile and appears to eventually promote establishment of successful antimicrobial treatment.

**Nucleotide sequence accession numbers**: The p14057A and p14057B sequences were submitted to GenBank under accession numbers KY296095 and KY296096, respectively.
